# Computational Insights Into Voltage Dependence of Polyamine Block in a Strong Inwardly Rectifying K^+^ Channel

**DOI:** 10.3389/fphar.2020.00721

**Published:** 2020-05-15

**Authors:** Xingyu Chen, Michael Bründl, Theres Friesacher, Anna Stary-Weinzinger

**Affiliations:** Department of Pharmacology and Toxicology, University of Vienna, Vienna, Austria

**Keywords:** molecular dynamics simulation, charge movement, ion displacement, inward rectification, Kir3.2, putrescine

## Abstract

Inwardly rectifying potassium (K_IR_) channels play important roles in controlling cellular excitability and K^+^ ion homeostasis. Under physiological conditions, K_IR_ channels allow large K^+^ influx at potentials negative to the equilibrium potential of K^+^ but permit little outward current at potentials positive to the equilibrium potential of K^+^, due to voltage dependent block of outward K^+^ flux by cytoplasmic polyamines. These polycationic molecules enter the K_IR_ channel pore from the intracellular side. They block K^+^ ion movement through the channel at depolarized potentials, thereby ensuring, for instance, the long plateau phase of the cardiac action potential. Key questions concerning how deeply these charged molecules migrate into the pore and how the steep voltage dependence arises remain unclear. Recent MD simulations on GIRK2 (=Kir3.2) crystal structures have provided unprecedented details concerning the conduction mechanism of a K_IR_ channel. Here, we use MD simulations with applied field to provide detailed insights into voltage dependent block of putrescine, using the conductive state of the strong inwardly rectifying K^+^ channel GIRK2 as starting point. Our µs long simulations elucidate details about binding sites of putrescine in the pore and suggest that voltage-dependent rectification arises from a dual mechanism.

## Introduction

Inwardly rectifying potassium (K_IR_) channels play important roles in controlling cellular excitability and K^+^ ion homeostasis. Under physiological conditions, K_IR_ channels pass inward currents more easily than outward currents due to voltage-dependent block by intracellular cations such as Mg^2+^ and polyamines (Hibino et al., 2010). These polycationic molecules enter the K_IR_ channel pore from the intracellular side and block K^+^ ion movement through the channel at depolarized potentials, thereby ensuring, for instance, the long plateau phase of the cardiac action potential (Nichols and Lee, 2018). The degree of inward rectification varies among the K_IR_ channel family. In strong inward rectifier channels, such as Kir2 and Kir3, very little outward current occurs at voltages positive to the K^+^ equilibrium (E_K_) potential. While in weak rectifier channels, e.g. Kir1 and Kir6 family members, conductance at voltages positive to E_K_ is reduced relative to inward currents. Intermediate rectification properties are found in Kir4.x channels (Hibino et al., 2010).

Atomic and near-atomic resolution structures of different K_IR_ channels, albeit without polyamines bound, have been solved mainly in the closed state, revealing a conserved architecture of the K_IR_ channel family. Further, the transmembrane domain (TMD) is conserved throughout the whole K^+^ channel superfamily (Doyle, 1998). It contains two transmembrane helices (M1 and M2), an extracellular turret region, a short P-helix and a selectivity filter region. A unique structural feature of K_IR_ channels is a long cytoplasmic domain (CTD), which contains another constriction, the so-called G-loop gate and extends the ion conduction pore to > 85 Å (Kuo et al., 2003).

Unlike voltage dependent gating of K_v_ channel proteins, voltage-dependent “inward rectification” in K_IR_ channels is not an intrinsic property. Voltage dependent rectification arises from voltage dependent channel blockade by cytoplasmic polycations, particularly polyamines, such as putrescine, spermine, and spermidine (Ficker et al., 1994; Lopatin et al., 1994; Fakler et al., 1995). Residues influencing rectification properties by polyamines have been identified in the CTD as well as the TMD. Two negatively charged residues in Kir2.1 (carrying I_K1_ current), E224 in the cytoplasm and D172, located in the transmembrane domain, have been shown to be critical for strong rectification. Interestingly, swapping residue D172 between strong and weak rectifier channels resulted in substantial transfer of rectification properties (Lopatin et al., 1994; Lu and MacKinnon, 1994; Stanfield et al., 1994; Wible et al., 1994). Due to the critical importance of this residue, this site has been referred to as rectification controller. Nevertheless, this position does not exert an all-or-nothing effect and surprisingly, channels such as Kir3.2, which exhibit relatively strong rectification lack this acidic residue (for alignment, see **Supplemental Figure 1**). Further, mutating D172 to N (Kir2.1) does weaken but not abolish polyamine binding (Wible et al., 1994; Yi et al., 2001; Guo et al., 2003). Importantly it has been shown that introducing a negative charge in the transmembrane domain is not position dependent (Kurata et al., 2004). This is in line with a computational study by the Roux group (Robertson et al., 2008), revealing that many of the residues influencing rectification interact with ions over very long distances up to 40 Å, likely affecting blocker energetics over the entire pore.

The CTD contributes to rectification to a lesser extent than the TMD. For example, interchanging the cytoplasmic domains of Kir2.1 and Kir1.1 resulted in partial transfer of rectification properties (Taglialatela et al., 1995). Mutagenesis identified two negatively charged residues in Kir2.1 (E224 and E299), being important determinants of rectification (Taglialatela et al., 1995; Kubo and Murata, 2001; Guo et al., 2003). Importantly, neutralization of these charges slowed the kinetics and reduced the affinity of spermine block (Taglialatela et al., 1995; Guo et al., 2003; Fujiwara and Kubo, 2006). However, mutations also changed the biophysics of the channel, complicating interpretation of the results.

Despite the existence of more and more crystal and cryo-EM structures, none of the different eukaryotic structures could be solved in the presence of polyamines so far, leaving important questions concerning the location of the polyamine binding site(s) within the K_IR_ channel pore open. Further, the nature of the charge movement associated with block and the origin of the steepness of the voltage dependence of these compounds, which roughly correlates with the number of charges of the polyamines remain unresolved.

Recent MD simulations on K_IR_ crystal structures have provided unprecedented details concerning the K^+^ conduction mechanism of Kir3.2 and 2.2 channels (Bernsteiner et al., 2019; Zangerl-Plessl et al., 2020) and can serve as excellent starting point to address inward rectification. Here, we perform multi-microsecond-timescale MD simulations with applied field to provide first atomistic insights into the voltage dependent block of putrescine (PUT^2+^), using the conductive state of the strong inwardly rectifying Kir3.2 channel as a starting point.

## Results

### Putrescine Block of Kir3.2 Channels Under Applied Field

**Figure 1:**
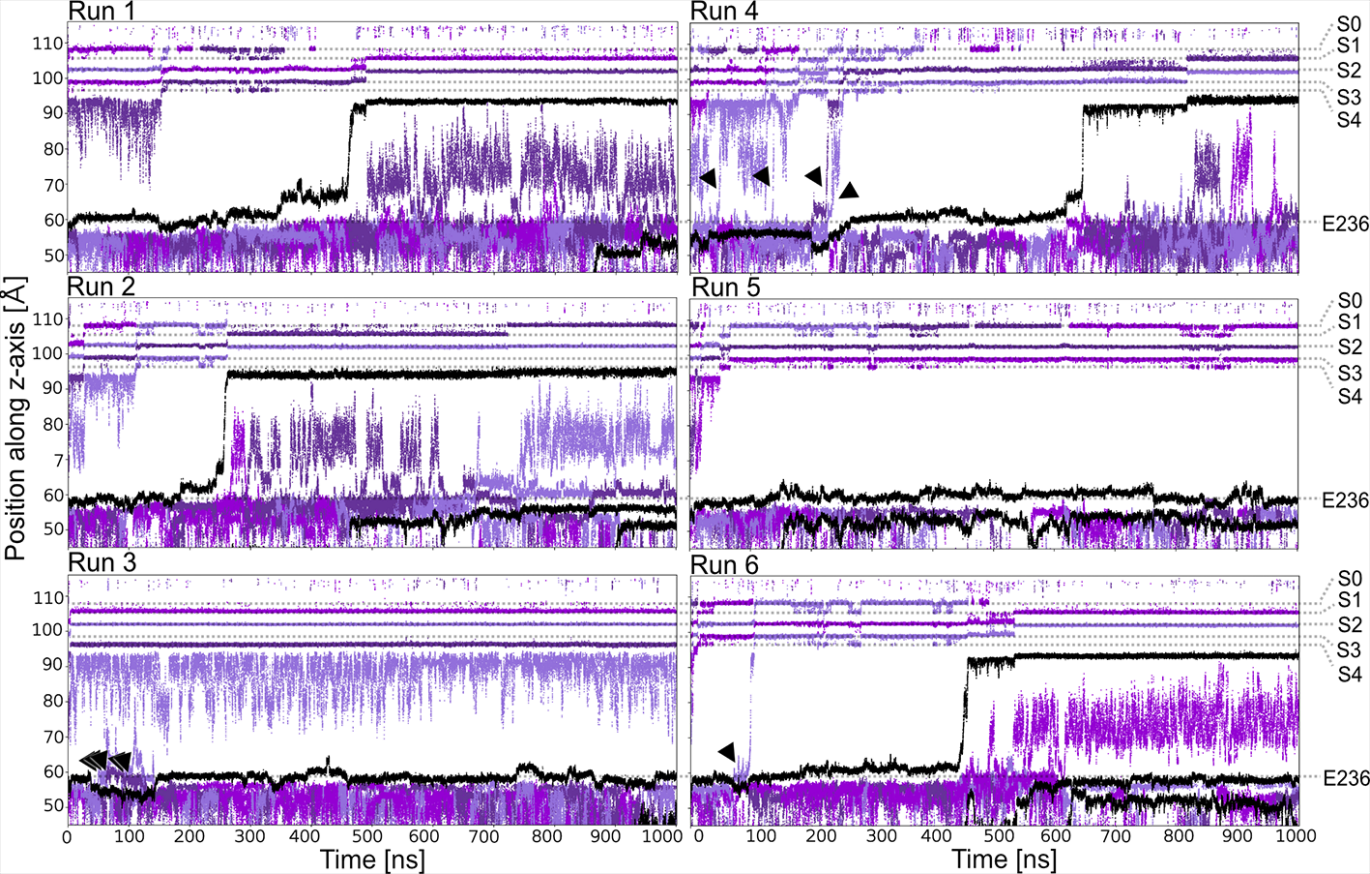
PUT^2+^ blockage of Kir3.2 channels under an applied field. Positions of K^+^ and PUT^2+^ molecules (center of mass) are plotted as function of time along the pore (z-axis). K^+^ ions are colored in different shades of purple and PUT^2+^ molecules are shown in black. Slippage of K^+^ ions is indicated with black arrows.

Starting from a conductive snapshot from a previous simulation (Kir3.2 also known as GIRK2), a bona-fide strong rectifier, we addressed the dynamics of the “shortest” polyamine putrescine (PUT^2+^), a 1,4- butanediamine, which has a net charge of +2 under physiological pH. **Figure 1** illustrates K^+^ and PUT^2+^ movement along the z-axis, monitored in six one µs atomistic simulations with an applied field of 40 mV/nm, which amounts to 580 mV. In four out of six runs, the molecule rapidly permeated from the initial binding site in the CTD to the selectivity filter (position S_C_ or S4). In the other two runs, PUT^2+^ remained close to the initial binding site in the CTD. Interestingly, in all except one run, at least a second, and in the case of run six, a third molecule entered from the cytoplasm to bind to the negatively charged residues in the CTD, mainly E236, revealing that the cytoplasmic pore can accommodate more than one blocker molecule. However, in none of the runs, more than one PUT^2+^ traversed up to the selectivity filter (SF). K^+^ permeation was effectively prevented only when the charged blocker reached its binding site at the SF but not when the polyamine was bound to the original binding region in the CTD. As illustrated in **Figure 1** in runs three, four and six, ions were observed to pass by PUT^2+^ when bound to the CTD (ion slippage examples are indicated by black arrows). This finding is in agreement with experimental data, suggesting a shallow blocked state for polyamines in the CTD (Kurata et al., 2007). The timescales for PUT^2+^ permeation to the SF varied from ~ 250 ns to ~650 ns, with the molecule remaining at its binding site for the rest of the simulation time. Binding to the CTD was reasonably stable when analyzing second and/or third PUT^2+^ molecules entering the CTD, with retention times of up to 850 ns, after accessing the binding site from the intracellular site. To analyze binding at these different sites in more detail, PUT^2+^ and K^+^ occupancies along the z-axis were plotted as function of time.

### Binding Sites of Putrescine in Kir3.2

**Figure 2:**
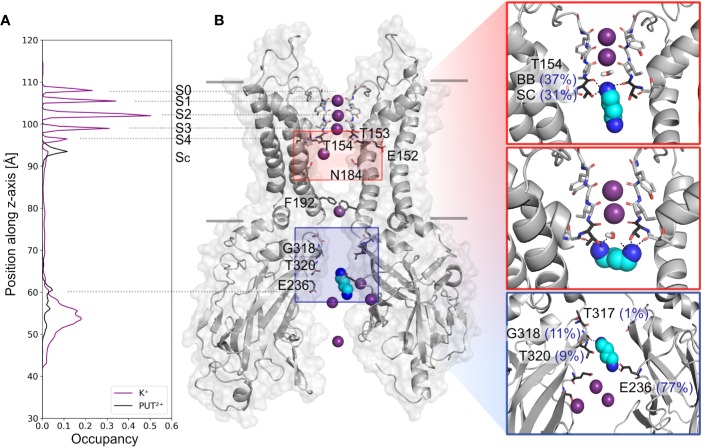
Putrescine occupancies and interactions with Kir3.2. **(A)** Histograms of K^+^ (purple line) and PUT^2+^ (black line) occupancies along the pore axis (z-axis) averaged from 4 µs MD simulations (runs 1, 2, 4, and 6). **(B)** Two opposing subunits of the equilibrated starting conformation of Kir3.2 (pdb: 3SYA), with K+ ions shown as purple spheres and PUT^2+^ shown as cyan spheres colored by atom name (nitrogen atoms colored in blue). Key residues for PUT^2+^ binding are labeled and shown in stick representation with oxygen atoms colored red and nitrogen atoms colored blue. Right panels: representative snapshots of PUT^2+^ binding sites at the SF (top and middle box) and the CTD (lower box) are shown, with hydrogen bond frequencies indicated in brackets (averaged over runs 1, 2, 4, and 6). BB, backbone; SC, sidechain.

Based on analyzing PUT^2+^ and K^+^ ion occupancies extracted from 4µs MD simulations (only from runs, where PUT^2+^ reached the SF binding site, compare **Figure 2B**), two binding areas emerged, one located close to E236 in the CTD, and a second, higher occupancy site close to T154 from the SF (**Figure 2A**). At the binding site in the CTD, frequent hydrogen bonds between the nitrogen atoms of PUT^2+^ and side-chains of E236 (equal to the known rectification-influencing residue E224 in Kir2.1) were observed. Less frequent hydrogen bond interactions to G318 and T320, close to the G-loop gate, were seen as well (see **Figure 2B**, right panel). When the molecule left this “shallow” blocking site, it rapidly passed the helix bundle-crossing gate (lined by residues F192) and the transmembrane cavity, without further intermediate binding sites. PUT^2+^ reached its final binding site at the bottom of the SF, where it oriented mainly perpendicular to the filter axis, which in one run was preceded by a horizontal orientation below the SF at site S_C_ (for representative snapshots, see **Figure 2B**, right panel). When PUT^2+^ was bound to the SF, it frequently formed hydrogen bonds with residue T154. Nitrogen atoms of the blocker interacted with the backbone carbonyl oxygen atoms as well as the side-chain of T154. Polyamine binding to the SF lead to displacement of a single K^+^ ion from the SF (**Figure 1** and **Supplemental Movies 1****–****4**). Comparison of ion occupancies between apo (taken from Bernsteiner et al., 2019) and holo runs revealed important differences. While, binding sites S2 and S3 were predominantly occupied in the apo runs, with significant K^+^ occupancy also observed in site S_C_, PUT^2+^ binding to the SF or at site S_C_ lead to displacement of ions from site S_C_, S4 or S3 upwards, expelling the uppermost K^+^ ion from the filter. Interestingly, a water molecule frequently occupied the site subsequent to PUT^2+^. K^+^ occupancy in site S2 was similar between apo and holo runs, while sites S1 and S2 revealed higher occupancy when PUT^2+^ was bound to the SF, as shown in **Supplemental Figure 2**.

### Simulations of Spermine Block (SPM^4+^)

**Figure 3:**
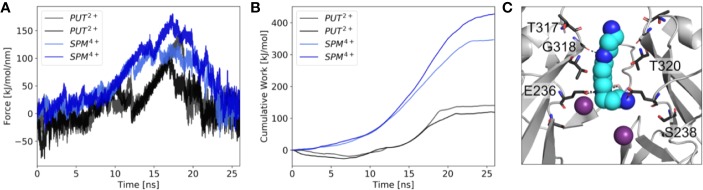
Force probe simulations of PUT^2+^ and SPM^4+^ pulling. **(A)** Force profiles of the polyamines, when pulled along the pore axis of Kir3.2 **(B)** work profiles for pulling PUT^2+^ (black) and SPM^4+^ (blue). **(C)** Representative snapshot of SPM^4+^ binding site at the CTD. K^+^ ions are shown as purple spheres, with interacting residues shown in stick representation.

Next, we performed µs MD simulations with spermine, a polyamine with a net charge of +4 under physiological pH. In contrast to simulations with PUT^2+^, SPM^4+^ did not traverse up towards the SF, but remained bound at the CTD site. As illustrated in **Figure 3C**, the compound mainly interacts with residues E236, T320, G318, and T317. Interestingly, in contrast to simulations with PUT^2+^ no slippage of K^+^ ions was observed in two times 1 µs runs. This suggests that SPM^4+^ completely blocks the channel already at this binding site. To probe if SPM^4+^ might be able to bind deep within the pore as observed for the much smaller PUT^2+^, we performed pulling simulations as reported previously for polymine block in a Kainate receptor (Brown et al., 2016). SPM^4+^ was pulled into the transmembrane cavity up to the position at the SF, observed for PUT^2+^ binding by applying a harmonic force along the z-axis, as detailed in the *Methods* section. Subsequently, the molecule was pulled in the reverse direction, towards the intracellular solvent and the work involved in this process calculated as described previously (Brown et al., 2016). Similar pulling simulations were also performed with PUT^2+^. As illustrated in **Figure 3**, the work needed for PUT^2+^ to traverse up to the SF binding site is much smaller compared to the larger SPM^4+^ molecule. Overall, similar trends have been reported for the Kainate receptor, even though we did not attempt to pull the polyamines through the selectivity filter. These data suggest that SPM^4+^ might be able to reach a binding site deep in the pore, below the SF, as observed for PUT^2+^. In the future, more extensive simulations will be required to assess SPM^4+^ block in Kir3.2 channels in more detail.

### Where Does the Steep Voltage-Dependence of Polyamine Block Come From?

**Figure 4:**
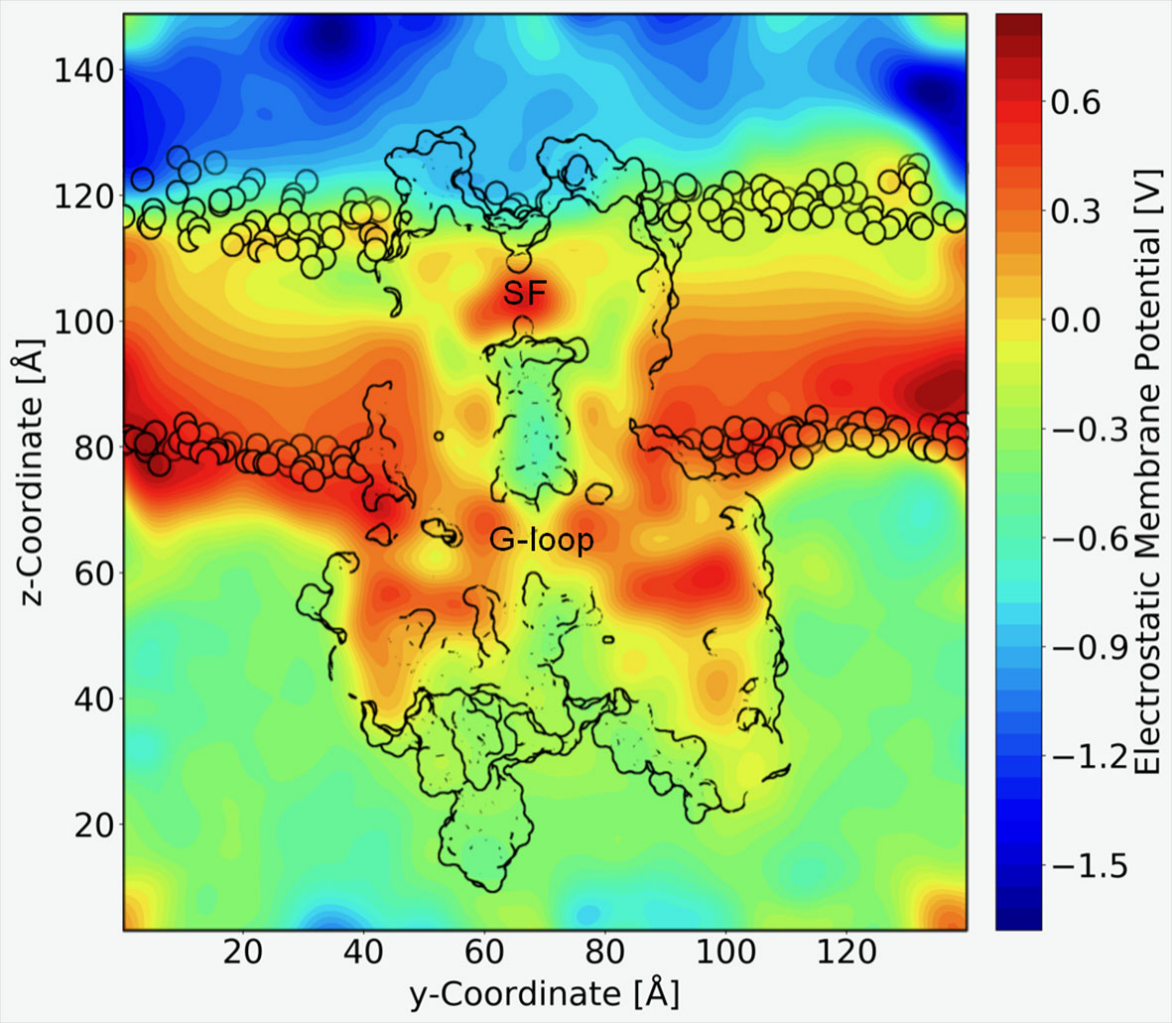
Electrostatic profile of Kir3.2. An external electric field of 0.5 V was introduced, as applied in free MD simulations. 21 snapshots from the trajectory were extracted and smoothed electrostatic potential grids with a resolution of 1 Å per grid point are shown. The overall shape of the Kir3.2 channels (side view), as well has phospholipid head groups are outlined in black. The selectivity filter (SF) and G-loop area are indicated.

A long standing question in the field concerns the steepness of the voltage dependence of polyamine block. It roughly correlates with the charge of the polyamine molecules itself, ranging from +2 (in the case of the diamine PUT^2+^) to +4 for SPM^4+^, which contains four nitrogen atoms (Nichols and Lee, 2018). To assess the contribution that blocker charge migration through the electric field might have on the steepness of the voltage dependence, we calculated the electrostatic profile along the Kir3.2 channel pore, extracting 21 frames from a 1µs trajectory, using the PME method as implemented in VMD (Aksimentiev and Schulten, 2005), as detailed in the *Methods* section. The electrostatic contour plots shown in **Figure 4**, reveal a previously unappreciated feature of the open state of Kir3.2. In accordance to the much wider pore of the MthK channel (27.3 Å, measured at position L95) (Jiang et al., 2002), the membrane electrostatic profile of the narrower Kir3.2 channel pore (Cα-Cα distances of 17.7 Å measured at F192, which is equivalent to position L95 in MthK) is predominantly focused on the SF region. Remarkably, however, there is also a significant fraction of the electric field focused on the narrow G-loop region (**Figure 4**). As previously reported, the region around residue G318, which forms the narrowest part of the G-loop gate, fluctuates between 5.9 to 6.8 Å, with oxygen atoms of G318 participating in K^+^ coordination (Bernsteiner et al., 2019). This suggests that the access resistance for a K^+^ ion diffusing between the cytoplasm and the SF might be higher in Kir3.2 compared to MthK. This agrees well with the much lower conductance rates in K_IR_ channels [~30 pS for Kir3.2 (Kofuji et al., 1996) vs. 170 pS for MthK channels (Li et al., 2007)]. Due to the narrower pore radius of the open K_IR_ channel, ions and other charged molecules such as PUT^2+^ experience a significant fraction of the membrane potential, when traversing from the CTD towards the SF. The electrostatic maps reveal that polyamine movement from the CTD towards a deep binding site below the SF might substantially contribute to the steepness of the voltage dependence of block (**Figure 3**). Further, partial binding to the SF, as observed for PUT^2+^ (**Figure 2**, left panel), contributes to voltage-dependence as well. This data also suggests that the electrical field distribution renders K^+^ conduction through the channel voltage-dependent. Indeed, such behavior has been previously reported by Spassova and Lu (Spassova and Lu, 1998).

## Discussion

In this work, we aimed to obtain first structural insights into the mechanism of PUT^2+^ and SPM^4+^ interactions, two naturally occurring polyamines, with the strong inward rectifier K^+^ channel Kir3.2. Polyamines are essential components in cells and bear ionisable amine moieties in their aliphatic chains. They play critical roles in protein synthesis, cell division and cell growth (Pegg, 2016; Handa et al., 2018). Their role in K_IR_ channel inhibition, due to pore block from the intracellular side was extensively studied (for detailed reviews see Nichols and Lopatin, 1997; Lu, 2004; Baronas and Kurata, 2014; Nichols and Lee, 2018), however, critical questions including the exact binding sites and the nature of the strong voltage-dependence are still unresolved.

We analysed blockade of the channel by performing multiple µs atomistic MD simulations on the GIRK2 crystal structure with an applied field to mimic a positive membrane potential, and performed pulling simulations to probe the work required for polyamines binding below the SF.

Two distinct, sequential polyamine binding sites were observed in our simulations with PUT^2+^, in agreement with kinetic modelling studies (Lopatin et al., 1995). A “shallow” binding site in the CTD was predicted, where the drug mainly interacted with a negatively charged ring, formed by E236, which is equivalent to E224 in Kir2.1 (**Figure 2B**, right panel, **Supplemental Figure 1**, for alignment), the best studied strong inward rectifier channel. This residue has previously been implicated in polyamine block. Neutralization has been shown to slow the rate of block, and reduce the apparent affinity of a second deep site block (Taglialatela et al., 1995; Kubo and Murata, 2001). In contrast, in simulations with SPM^4+^, only the CTD binding site was observed in the 1 µs runs, due to limited sampling. Thus, by applying enhanced sampling (pulling simulations) on the polyamines, we were able to sample binding of SPM^4+^ at the “deep” binding site, below the SF. Binding of this larger polyamine required three times more work, compared to the much smaller PUT^2+^, suggesting that it is much easier for PUT^2+^ to move up to the SF site, compared to SPM^4+^. A similar trend has previously been reported for Kainate receptor polyamine interactions (Brown et al., 2016).

Remarkably, our simulations revealed that PUT^2+^ could not completely inhibit ion conduction when interacting with the negatively charged residues in the CTD, since “slippage” of K^+^ ions past the bound polyamine upwards into the transmembrane region was observed in several runs (**Figure 1**). This observation agrees with previous suggestions of incomplete block at the CTD, based on functional studies on Kir2.1 (Xu et al., 2009). Interestingly, no slippage was observed, when SPM^4+^ was bound to the same site in the CTD. However, longer sampling will be required to address, if this difference indeed holds true on longer simulation time scales.

Further, MD simulations revealed that PUT^2+^ could move through the electric field up to a steep binding site, at the SF where it stably interacted with T154, located at the base of the filter. This residue has not been tested experimentally for polyamine block, but it has been implicated in high-affinity block of Ba^2+^ in Kir2.1 channels (Hsieh et al., 2015). Support for blocker migration deep into the pore comes from blocker protection experiments with MTS reagents, suggesting that polyamines might bind above the rectification controller position D172 (Kurata et al., 2008).

Another important observation of our study is that PUT^2+^ migration to the SF binding site is coupled with displacement of a permeant K^+^ ion. Such a scenario has recently been observed in CP-AMPAR channels, where polyamines with different tail length have been analyzed, using cryo-EM. Structures with short polyamines reveal that these molecules partially protrude into the SF, while longer molecules bind deep within the SF. The cryo-EM structure demonstrates that a combination of blocker entry into the filter and ion displacement can lead to steep voltage dependence (Twomey et al., 2018). Our simulations suggest a similar mechanism for inward rectifier channels. Further, such a mechanism has been previously envisioned, based on experimental observation, showing that the blocker dependence on the membrane voltage varied as a function of extracellular K^+^ concentration (Spassova and Lu, 1998; Whorton and MacKinnon, 2011).

An important, previously largely unappreciated finding of our study concerns the nature of the electric field. In contrast to previous expectations, based on calculations of K^+^ channel pores with wider gate diameters (e.g. Jiang et al., 2002), the membrane potential does not only drop over the selectivity filter, but there is a significant voltage drop over the G-loop area, as illustrated in **Figure 4**. Based on these qualitative calculations, polyamine binding to the CTD site is likely to depend on the prevailing voltage (without ion displacement from the SF), due to molecules traversing a fraction of the membrane potential, when passing through the G-loop gate area.

In addition, our simulations consistently predict that binding of PUT^2+^ to the SF site leads to outward displacement of a K^+^ ion (**Figure 1** and **Supplemental Movies**). These predictions agree very well with functional studies, supporting sequential binding with weak and strong voltage dependence (Lopatin et al., 1995; Shin and Lu, 2005; Kurata et al., 2007).

The predicted differences in electrostatic potential along the pore further suggest that the access resistance for K^+^ ions diffusing between the cytoplasm and the SF is higher in Kir3 channels, compared to ion channels with wider open pores such as MthK. This is in line with the different conductance rates reported for these channels, which amount to ~ 30 pS in Kir3.2 channels (Kofuji et al., 1996), but are much higher in MthK channels, where values of ~ 200 pS have been published (Li et al., 2007). The electrostatic data further suggests that the electrical field distribution should render K^+^ conduction voltage dependent, which is in agreement with previous reports by Spassova and Lu (1998).

A clear limitation of our study is the fact that so far we tested only two polyamines PUT^2+^ and SPM^4+^ on an “atypical” strong inward rectifier channel that has been poorly studied in the homotetrameric form. Studies investigating polyamine block in Kir3 channels have almost exclusively focused on the heterotetrameric versions composed of Kir3.1 and Kir3.4 (carrying I_KACh_ currents) (Yamada and Kurachi, 1995; Salazar-Fajardo et al., 2018). However, strong inward rectification was confirmed for the crystal structure construct of Kir3.2 (Whorton and MacKinnon, 2011), justifying the use of this construct to study the mechanisms of inward rectification. Further, the open state structure of the extremely well characterized Kir2.1 channel is still not available.

Given the very recent success to crystallize Kir2.2 channels in an open, conductive conformation (Zangerl-Plessl et al., 2020) future studies will need to be performed to address polyamine block in the classic strong rectifier Kir2 family. Comparison of conductance studies on Kir3.2 and Kir2.2 channels reveal very interesting differences, which might affect polyamine block. While the inner (transmembrane) cavity of Kir3.2 contains on average only one K^+^ ion (Bernsteiner et al., 2019), simulations on Kir2.2 channels revealed that the inner cavity in these channels can harbour up to five K^+^ ions, located close to the acidic charges of the rectification controller, which is lacking in Kir3.2 channels. Thus, it might be envisioned that perhaps polyamine entry into the inner cavity would lead to different ion displacement between these different channels. Further, the diameter of the G-loop region is quite different in the two Kir families. While no Kir2.2 structure showing a narrow G-loop region has been reported so far, various constricted conformations have been reported for Kir3 structures. Our recent simulations on WT Kir3.2 channels (Bernsteiner et al., 2019) vs. Kir2.2 mutant channels (Zangerl-Plessl et al., 2020) support a different behaviour of this region in the two channels. Future studies will be necessary to address similarities and differences inward rectification mechanisms in other Kir channels in detail.

Summarizing our simulations predict that blocker amines can indeed interact with the SF, and that voltage dependence of inward rectification arises in part from the movement of the blocker through the electric field and energetic coupling between blocker binding and permeating K^+^ ions.

## Material and Methods

### Molecular Dynamics Simulations

Gromacs (version 5.1.2) (Abraham et al., 2015) was used to perform molecular dynamics simulations, with the conductive state of the Kir3.2 channel, embedded in a lipid bilayer consisting of 588 POPC (1-palmitoyl-2-oleoyl-sn-glycero-3-phosphocholine) lipids, taken from Bernsteiner et al., (Bernsteiner et al., 2019). PIP_2_ parameters were taken from our previous work (Lee et al., 2016). Berger lipid parameters (Berger et al, 1997) were used for the POPC lipids, the amber99sb forcefield (Hornak et al., 2006) for the protein, and corrected monovalent Lennard-Jones parameters (Joung and Cheatham, 2008) were used for K^+^ ions. After neutralization with K^+^ ions, 150 mM KCl was added to the simulation system. For Lennard-Jones and electrostatic interactions, a cut-off of 1.0 nm was employed, with long-range electrostatic interactions calculated with the Particle-Mesh Ewald algorithm (Essmann et al., 1995). Bonds were constrained using the LINCS algorithm (Hess et al., 1997), allowing for an integration timestep of 2 fs. Temperature was coupled to 310 K using the v-rescale thermostat (Bussi et al., 2007), with a coupling constant of 0.1 ps. The pressure was kept constant semi-isotropically at 1 bar by the Parrinello-Rahman (Parrinello and Rahman, 1981) barostat (τ = 2 ps). Putrescine was parameterized using the Hartree-Fock geometry optimization with the 6–31G* basis set in Gaussian (Frisch et al, 2016) and the force field parameters are obtained from AmberTools (Case et al., 2018). All amine nitrogen atoms were treated as charged. Six times one µs runs with an applied electric field of 40 mV nm^−1^ along the z-axis of the simulation box were performed. With a z-axis box length of ∼14.5 nm, this resulted in transmembrane potentials of 580 mV (Treptow et al., 2004; Roux, 2008). 20 putrescine molecules were added to the solvent, with one molecule initially placed at the entrance of the CTD, as shown in **Figure 2B** (left panel), using gromacs tools. PUT^2+^ and K^+^ occupancies were calculated as described previously (Bernsteiner et al., 2019).

### Force Probe (Steered) MD Simulations

The work needed to bind and unbind PUT^2+^ and SPM^4+^ from the Kir3.2 channel was assessed using force probe MD simulations. Polyamines were placed perpendicular to the z-axis and pulled into the transmembrane cavity towards position T154 (bottom of SF) and in the reverse direction towards the cytoplasm with a harmonic force of 100 kJ mol^-1^ nm^-2^ and a velocity of 0.25 nm ns^-1^. The work was calculated from the force profiles as previously described (Brown et al., 2016) using the following equation:

$$W\left(t\right)=v\int_{0}^{t}f(t)dt$$

where v is the constant velocity, f(t) is the force at a specific time step given in the force probe output and dt is the time step between data points (100 fs).

### Electrostatic Profile Calculations

The electrostatic profile along the Kir3.2 channel was calculated based on a 1 µs trajectory of a conductive WT Kir3.2 run taken from Bernsteiner et al., 2019. In total, 21 frames were extracted (one frame each 50 ns) and aligned at the selectivity filter backbone. We subsequently applied the VMD Plugin PME Electrostatics to each of the frames, which generates smoothed electrostatic potential grids by evaluating the reciprocal sum of the smooth particle-mesh Ewald method (PME) (Aksimentiev and Schulten, 2005; Treptow et al., 2009). By setting the size of the three dimensional grid to 144x144x160 points, we established a resolution of 1 Å per grid point. The Ewald factor was set to 0.25. Charge information was included by converting the PDBs to PSF files with Amber18 (Case et al., 2018) based on the Amber99sb force field with Berger lipids (see MD methods). Following the protocol for grid manipulation of the Aksimentiev group, an external electric field of 0.5 V was introduced to the system. The electrostatic profile was averaged over the 21 frames and plotted by extracting the y-z plane of the grid, which slices through the selectivity filter. A python script was kindly provided by Kumar Sarthak from the Aksimentiev group.

## Data Availability Statement

The datasets presented in this study can be found in online repositories. The names of the repository and accession number(s) are: Zenodo, http://doi.org/10.5281/zenodo.3760965.

## Author Contributions

AS-W designed the study. XC and MB performed simulations. XC, MB, TF, and AS-W analyzed the data. AS-W wrote the paper, which was edited by XC, MB, and TF.

## Funding

This work was supported by the doctoral program “Molecular drug targets” W1232, from the Austrian Science Fund (FWF; http://www.fwf.ac.at).

## Conflict of Interest

The authors declare that the research was conducted in the absence of any commercial or financial relationships that could be construed as a potential conflict of interest.
