# Supplementary material for: Computational Insights Into Voltage Dependence of Polyamine Block in a Strong Inwardly Rectifying K+ Channel
Source: Front Pharmacol. 2020 May 15;11:721. doi: 10.3389/fphar.2020.00721 (PMC7243266; doi:10.3389/fphar.2020.00721)
Supplement: Supplementary file 1 [file DataSheet_1.docx]

Supplementary Material

**Supplementary Figures**


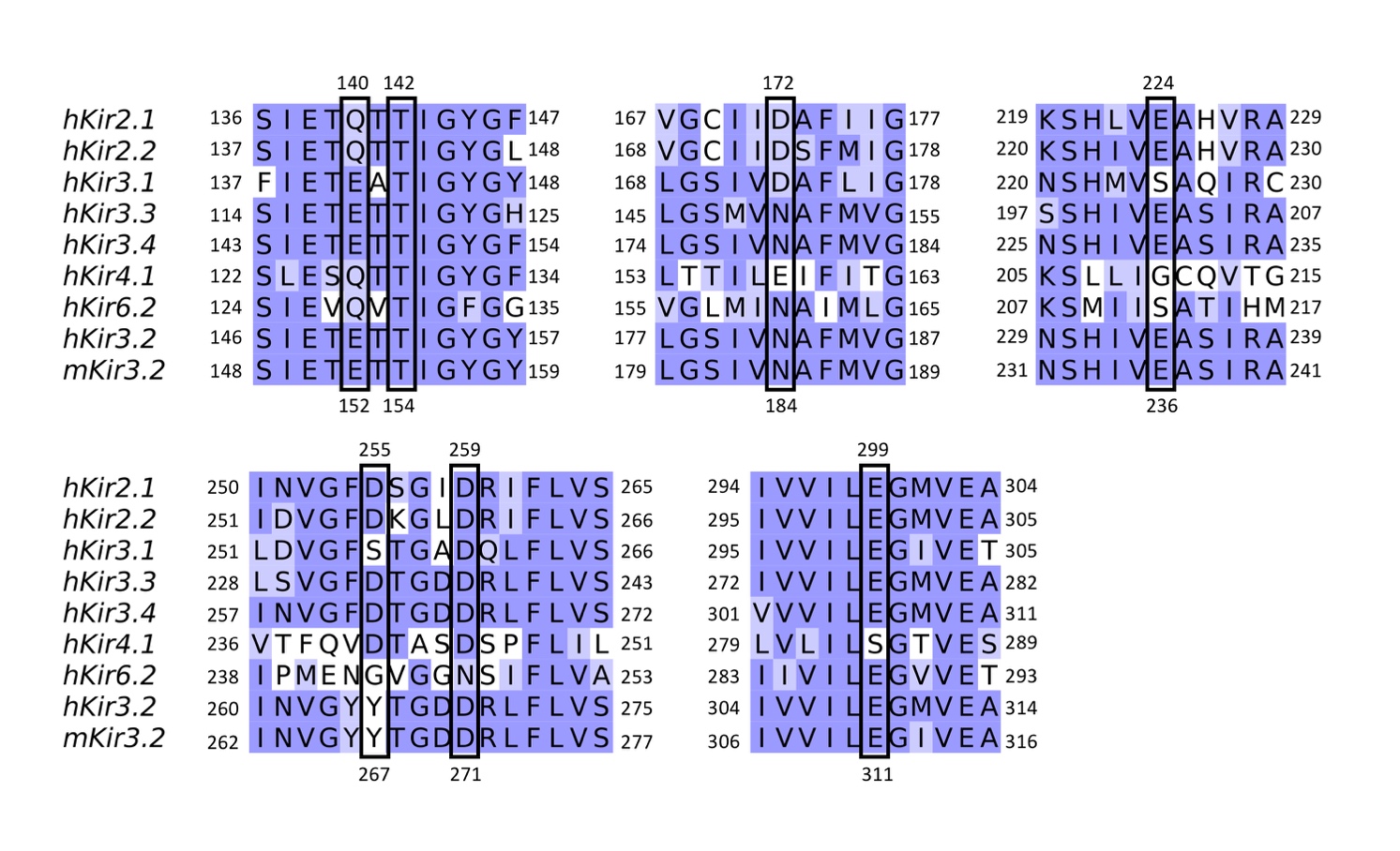


**Supplementary Figure 1. Sequence alignment of human Kir family and mouse Kir3.2 channels**. Key residues for putrescine binding are highlighted with black frames. h: Homo sapiens (Human); m: Mus musculus (Mouse).


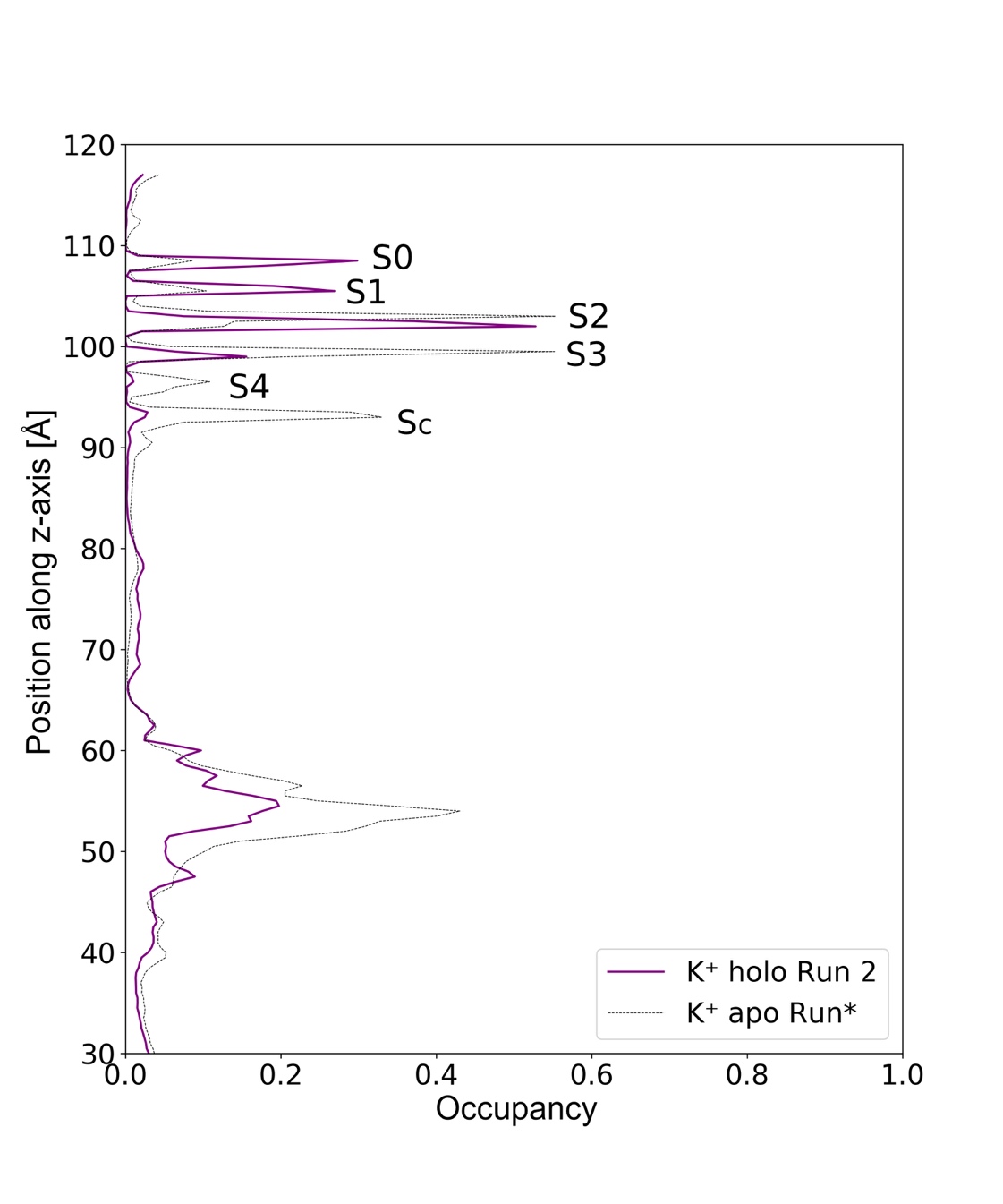


**Supplementary Figure 2.** The comparison of K^+^ occupancies in the apo (Bernsteiner et al., 2019) and holo (PUT^2+^ presented) Kir3.2 channels from MD simulations.

**Movies 1 - 4**

Dynamics of PUT^2+^ and K^+^ ions in four times 1 µs MD simulations, with 2 opposing subunits shown as in Figure 2B, middle panel.

**References**

Bernsteiner, H., Zangerl-Plessl, E.-M., Chen, X., and Stary-Weinzinger, A. (2019). Conduction through a narrow inward-rectifier K + channel pore. *J. Gen. Physiol.* 151, jgp.201912359. doi:10.1085/jgp.201912359.
